# Characterizing the cirri and gut microbiomes of the intertidal barnacle *Semibalanus balanoides*

**DOI:** 10.1186/s42523-020-00058-0

**Published:** 2020-11-13

**Authors:** Bianca R. P. Brown, Joaquin C. B. Nunez, David M. Rand

**Affiliations:** 1 Department of Ecology and Evolutionary Biology, Brown University, 80 Waterman St., Providence, RI 02912 USA; 2 Institute at Brown for Environment and Society, Brown University, 85 Waterman St., Providence, RI 02912 USA; 3 Department of Biology, University of Virginia, 485 McCormick Road, Charlottesville, VA 22904 USA

**Keywords:** Barnacles, Intertidal, Microbiome, *Semibalanus balanoides*, 16S rRNA gene, Rocky shore

## Abstract

**Background:**

Natural populations inhabiting the rocky intertidal experience multiple ecological stressors and provide an opportunity to investigate how environmental differences influence microbiomes over small geographical scales. However, very few microbiome studies focus on animals that inhabit the intertidal. In this study, we investigate the microbiome of the intertidal barnacle *Semibalanus balanoides*. We first describe the microbiome of two body tissues: the feeding appendages, or cirri, and the gut. Next, we examine whether there are differences between the microbiome of each body tissue of barnacles collected from the thermally extreme microhabitats of the rocky shores’ upper and lower tidal zones.

**Results:**

Overall, the microbiome of *S. balanoides* consisted of 18 phyla from 408 genera. Our results showed that although cirri and gut microbiomes shared a portion of their amplicon sequence variants (ASVs), the microbiome of each body tissue was distinct. Over 80% of the ASVs found in the cirri were also found in the gut, and 44% of the ASVs found in the gut were also found in the cirri. Notably, the gut microbiome was not a subset of the cirri microbiome. Additionally, we identified that the cirri microbiome was responsive to microhabitat differences.

**Conclusion:**

Results from this study indicate that *S. balanoides* maintains distinct microbiomes in its cirri and gut tissues, and that the gut microbiome is more stable than the cirri microbiome between the extremes of the intertidal.

**Supplementary Information:**

The online version contains supplementary material available at 10.1186/s42523-020-00058-0.

## Background

Like all animals, marine invertebrates have evolved in close association with microbes, forming symbiotic relationships with microorganisms known as the microbiome [1–5]. The microbiome provides several functions to its host, including nutrient acquisition and protection from pathogens, and without a microbiome some species show reductions in development rates and other measures of organismal fitness [6, 7]. Yet, the strength of the symbiotic relationship between hosts and their microbiomes, and the reliance of hosts on their microbiome functions, varies across species [8, 9]. Despite our growing knowledge of host-microbiome interactions and the factors that shape them across a range of species, there is still a lack of understanding of how the microbiome and its functions are maintained across space and time in many sessile marine invertebrates, particularly those that inhabit intertidal environments.

Sessile invertebrates have unique life-history traits that shape their microbiome. Like all invertebrates, sessile invertebrates only possess an innate immune system which lacks immunological memory, whereas vertebrates have both innate and adaptive immune systems. For marine invertebrates, hemolymph is a critical component of the immunological response [10]. Hemolymph consists of hemocytes that perform defense functions such as phagocytosis, and the production of reactive oxygen species to rid the host of pathogens. Understanding how invertebrates are able to distinguish pathogens from commensals, and how the hemolymph microbiome shifts under varying stresses, have been underlying questions of many microbiome studies [5, 11, 12].

In addition to understanding the link between the microbiome and the innate immune system, sessile invertebrates can expand our understanding of how diet shapes microbiome composition. Diet is an important factor in shaping the microbiome [13]. Most sessile invertebrates filter feed (i.e., a feeding strategy that often requires muscle or ciliary action to capture particles floating around from ambient fluid currents in the water column) [14, 15]. The sessile lifestyle, along with the filter-feeding strategy, offers distinct modes of understanding how diet and behaviors shape the microbiome compared to commonly studied mobile (filter-feeding and non-filter feeding) invertebrates and vertebrates [8, 13, 16, 17].

Finally, sessile invertebrates can offer insight into how environmental shifts influence the microbiome at local scales. Unlike vertebrates, sessile invertebrates cannot escape the stresses associated with their local environments. The combination of being ectothermic and immobile make sessile invertebrates highly susceptible to environmental changes [18–20]. Thus, studying the microbiome of sessile invertebrates can potentially signal when local conditions are changing, as well as demonstrate how those changes influence microbiome and host.

**Fig. 1:**
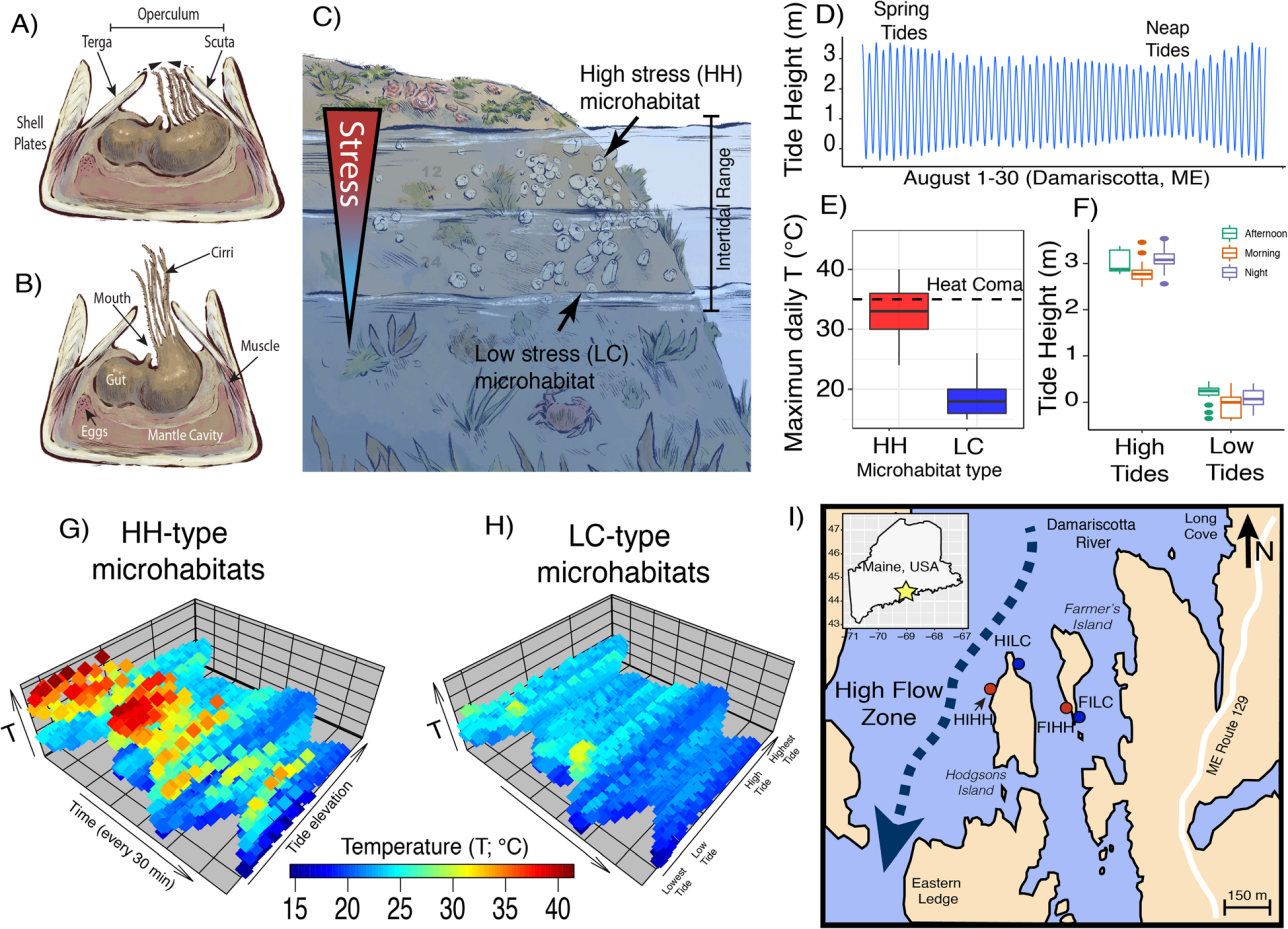
Model of barnacle, its surrounding environment, and map of collection site. **a** Diagram of a barnacle with cirri retracted and the operculum closed which occurs during periods of emersion. **b** Diagram of a barnacle with extended cirri during filter feeding. **c** Model of intertidal stress across the rocky intertidal. **d** Measurements of tidal height for the month of August 2015 in Damariscotta, ME (NOAA tide station 8416731). **e** Measurements of maximum daily temperatures across collection sites for sites high temperature sites (High Hot-HH) and low temperature sites (Low Cold-LC) (shown in panel **c**). Data was collected in the summer of 1994 and published by Schmidt and Rand [23]. The horizontal dashed line indicates the heat coma point. **f** Range of tidal height for the high and low tides in August 2015 at the Damariscotta River, ME at mornings, afternoons, and nights. **g** High-resolution temperature profile of HH habitats as a function of tidal range and summertime in Rhode Island. Data was collected in the summer of 2018 and published by Nunez et al. [24, 25]. The temperature axes show temperatures collected every 30 min. The time axis shows times from July to August. The tidal range axis represents the tidal height at the moment of temperature collection (NOAA tide station 8452660, Newport, RI). The oscillations in the tidal range amplitude are produced by the lunar cycle, and the points of maximum thermal stress coincide with the lowest low tides. **h** Same as G but for LC habitats. **i** Map of the Damariscotta River in Maine with sampling localities indicated. Barnacles were collected from Hodgson’s Island (HI) and Farmer’s Island (FI). There are four sites: Farmers Island Hot High (FIHH), Farmers Island Low Cold (FILC), Hodgsons Island High Hot (HIHH), and Hodgsons Island Low Cold (HILC). Red dots indicate upper tidal habitats with high sun exposure. Blue dots indicate lower tidal habitats with low sun exposure

Natural populations of the intertidal northern acorn barnacle (*Semibalanus balanoides*) provide a robust system in which to investigate changes in microbiome composition across highly heterogeneous environments. For decades, *S. balanoides* has been studied as a model of ecological zonation [18, 21–25]. The life cycle of *S. balanoides* consists of a yearly mating window (August–September) during which barnacles only mate with individuals settled in their immediate proximity, followed by brooding (September–December), and subsequent hatching and release during the late winter plankton blooms [26, 27]. At this stage, larvae have high dispersal capabilities, swimming freely in the water column for up to 5 weeks and up to 100 km from the point of spawning [28]. After settlement has occurred, barnacles cement themselves to the substrate and grow a calcareous shell [15] (Fig. 1a and b). *S. balanoides* recruits to a wide range of microhabitats, extending from the upper to the lower intertidal zone (Fig. 1c). While spatially adjacent, these microhabitats pose drastically different ecological challenges to individuals. For instance, Schmidt and Rand [23] showed that barnacles recruiting near the upper edge of the intertidal zone endure increased levels of thermal stress that leads to higher occurrences of desiccation relative to counterparts in the lower edge of the intertidal (Fig. 1d). During the summer, temperatures in the upper intertidal can regularly surpass 35 °C, a point at which heat-induced coma has been observed [19, 21]. At this temperature, the barnacle can no longer perform normal activities [19]. Moreover, during particularly hot days, temperatures can rise above 37 °C, a point at which mass mortality has been recorded in experimentally reared populations [21]. In addition, Nunez et al. [25] showed that upper intertidal sites experience a higher degree of temperature variation year-round, relative to the thermally homogeneous lower intertidal sites. These differences are most apparent during the summer low tides (Fig. 1g and h). Aside from abiotic factors, the stress of the upper intertidal zone can be modulated by both biotic and abiotic interactions, as well as by geographical location [29]. Along the Atlantic coasts, barnacles are sometimes found in close association with algae, and algal cover can offer protection from the sun and aid in keeping barnacles hydrated [18]. Depending on the abiotic and biotic conditions within the upper tidal zone, individuals can experience physiological challenges such as reduced feeding, concomitant with increased waste accumulation and heat stress that increases mortality, especially during the summer months in sun-exposed areas without algal cover [18, 22]. The resulting stress gradient experienced by *S. balanoides* in the intertidal provides a natural laboratory in which to investigate how the microbiome changes in response to stress fluctuations in a natural population.

Differences in natural selection at ecologically important genes across the intertidal [24, 25], as well as different profiles of environmental stress, and biotic interactions across intertidal microhabitats, provide reasons to hypothesize that *S. balanoides* may show different microbiome compositions across thermal gradients. Studies in species with similar life habits, such as oysters and mussels, have shown that shifts in temperature result in changes in relative abundance of microbes [30]. In pacific oysters, hemolymph microbiome diversity, which as previously stated plays a critical role in marine invertebrate immunological response, increases with higher seawater temperatures [12]. Moreover, studies investigating other intertidal species such as sponge (*Hymeniacidon heliophila*) and algal (*Corallina officinalis*) species have observed differences in microbiome composition among intertidal and subtidal conspecifics [31, 32].

This study serves two purposes. First, we characterize the microbiome composition and function of the barnacle feeding appendages (i.e., the cirri) and gut of *S. balanoides* (Fig. 1b) using 16S rRNA amplicon sequencing. Second, we ask whether intertidal microhabitat, or an individual barnacle’s position relative to the upper or lower edges of the intertidal range, (Fig. 1c), influences the microbiome. We conduct this analysis across two islands in the Damariscotta river in Maine, USA (Fig. 1i). Barnacles are facultative filter feeders. They can either actively drive water through their cirri pushing particles to their mouth or passively collect food from ambient currents [33]. As such, the cirral appendages are constantly exposed to the environment. The gut, on the other hand, is an internal organ protected inside the barnacle prosoma. Given that barnacles are known to discriminate among food items [15], we hypothesized that the microbiome of the gut would be similar to, or a subset of, the microbiome of the cirri. That is, it would be driven by the subset of microbes found in particles “accepted” during feeding. Consequently, since the cirri are more exposed to the external environments, we also hypothesized that the microbiome of the cirri would be more labile relative to the gut, across microhabitats. The combination of the two dimensions of our study, the intertidal stress gradient, and the different body parts, affords the opportunity to investigate the effects of thermal stress on the lability and composition of microbiomes. As such, understanding the microbiome of *S. balanoides* provides novel insights into how natural environmental stressors influence host-microbiome interactions.

## Results

### Quality check and filtering

We processed gut and cirri samples from 12 individual barnacles collected from four microhabitat: three Farmers Island Hot High (FIHH), three Farmers Island Low Cold (FILC), three Hodgsons Island High Hot (HIHH), and three Hodgsons Island Low Cold (HILC). A total of 24 samples were collected and processed (*N* = 12 gut and *N* = 12 cirri; Table 1). At the beginning of the analysis, we identified 13,226,321 (mean = 544,887.27, SD = 122,763.42) sequences across 24 samples (Supplementary Table 1). After processing with DADA2 [34], removing contaminants, unwanted sequences (e.g. sequences characterized as mitochondrial, eukaryotic, archaeal, and chloroplast), and mock samples there was a total of 904,733 (mean = 72,378.64, SD = 42,206.81) bacterial amplicon sequence variants (ASVs). On average, cirri samples had lower ASV abundance than gut samples (mean cirri = 19,320.40, SD cirri = 11,923.45; mean gut = 56,074, SD gut = 53,346). However, when we removed samples with ASVs < 5000, a gut sample had the lowest number of ASVs (10,372 ASVs). Samples were rarefied to sample with the smallest ASV count (10,372). Plateauing of rarefaction curves confirmed that each sample had a good representation of the ASVs within the bacterial community (Supplementary Figure 1). After rarefying, there were 217,812 ASVs, of which 1477 were unique across all samples. Additionally, there were no singletons in our dataset. At the end of processing 10 cirri and 11 gut samples remained (Table 1).

**Table 1:** Sample size for each body tissues and associated microhabitats. Twenty-four samples were processed from 12 barnacles. Numbers in parentheses indicate sample size after filtering and processing of sequences

	Farmers Island (FI)	Hodgsons Island (HI)
**High and Hot (HH)** **Cirri**	3 (1)	3
**Low and Cold (LC)** **Cirri**	3	3
**High and Hot (HH)** **Gut**	3	3 (2)
**Low and Cold (LC)** **Gut**	3	3

Dissection and DNA extraction

### Gut and cirri microbiome composition and core microbiome

**Fig. 2:**
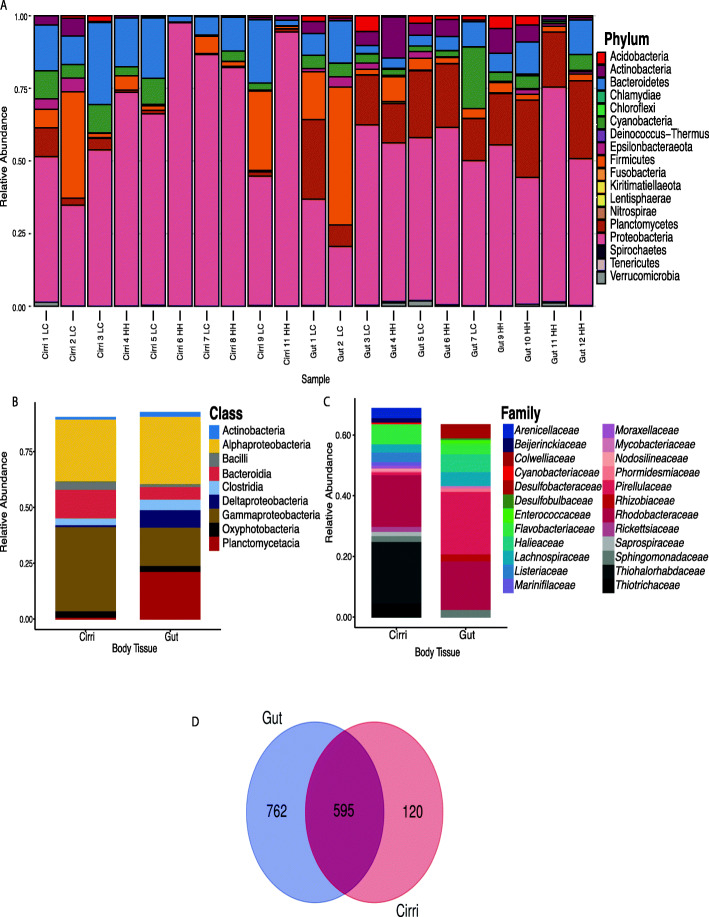
Relative abundance of ASVs. **a** Relative abundance of ASVs of each sample at the phylum level. **b** Mean relative abundance (> 0.05) of ASV at the Class and **c** family level of gut and cirri. **d** Venn diagram of ASVs unique to and shared between cirri and gut

When all 1477 ASVs were taken into account, 762 ASVs were unique to the gut, 120 ASVs to the cirri, and 595 ASVs were shared between cirri and gut (Fig. 2d). Both cirri and gut shared the same top six Phyla with the highest mean relative abundance: Proteobacteria (cirri 68%; gut 51%), Bacteroidetes (cirri 13%; gut 7%), Firmicutes (cirri 9%; gut 9%), Cyanobacteria (cirri 4%; gut 5%), Planctomycetes (cirri 2%; gut 20%), and Actinobacteria (cirri 1%; gut 5%) (Fig. 2a, Supplementary Table 2). Actinobacteria and Planctomycetes were significantly higher in abundance in the gut than in the cirri (Supplementary Figure 2). Other top phyla (Proteobacteria, Bacteroidetes, Firmicutes, and Cyanobacteria) were present in similar abundance between body types (Supplementary Figure 2). The top classes observed within the cirri were Gammaproteobacteria (38%), Alphaproteobacteria (28%), Bacteroidia (13%), Bacilli (5%), and Oxyphotobacteria (4%) (Supplementary Table 2). Within the gut, the top classes observed were Alphaproteobacteria (27%), Plactomcycetacia (19%), Gammaproteobacteria (16%), Deltaproteobacteria (8%), and Bacteroidia (6%) (Fig. 2b, Supplementary Table 2).

With respect to the core microbiome community, we found no ASV that was shared among all our samples (cirri and gut), nor was there an ASV shared across all gut or cirri samples. Therefore, we defined core microbiome as ASVs present in at least 70% of the sample type of interest. At 70% prevalence, the cirri core microbiome consisted of nine shared ASVs, and gut core microbiome had 71 shared ASVs (Supplementary Figure 3, Supplementary Table 3). Shared ASVs from the cirri core microbiome constituted 25% of the mean relative abundance of cirri samples (Supplementary Figure 3). Shared ASVs from the gut core microbiome represented 15% of the mean relative abundance of gut samples (Supplementary Figure 3). The nine shared ASVs that made up the cirri core microbiome all belonged to Protobacteria. Of the shared ASVs associated with cirri samples, the genera *Granulosicouccus* (10%) and *Roseobacter* (6%) had the highest mean relative abundance. The remaining shared ASVs found among cirri samples belonged to *Methylobacterium*, *Leucothrix*, *Aliiroseovarius*, *Loktanella*, *Octadebacter*, and MD4–55. The gut core microbiome ASVs were from Proteobacteria (43), followed by Planctomycetes (14), Acidobacteria (6), Episilonacteraeota (3), Cyanobacteria (2), Bacteroidetes (2), and Firmicutes (1). Shared ASVs that formed the gut core microbiome were each present at < 1% mean relative abundance. The genera of the shared ASVs with the highest relative mean abundance were *Aliiroseovarius* and *Desulforsarcina* with 0.7%.

### Microbiome diversity and composition differences across body tissues

**Fig. 3:**
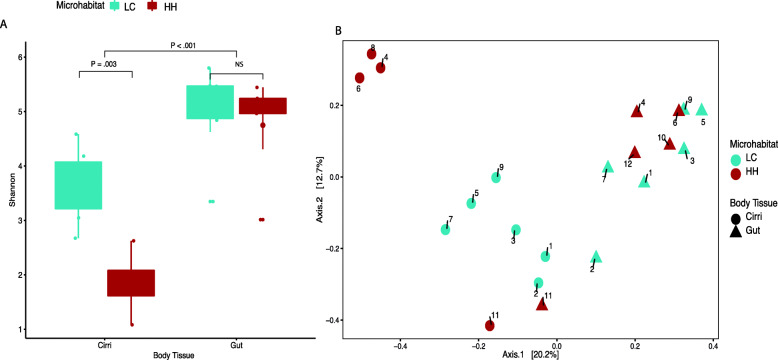
Microbiome diversity of cirri and gut within the intertidal and across locations of 21 samples (10 cirri;11 gut). **a** Shannon diversity index of cirri and gut samples. **b** PCoA of Bray–Curtis dissimilarity distances among samples. Numbers represent individuals. Gut and cirri samples with the same number are from the same host

**Fig. 4:**
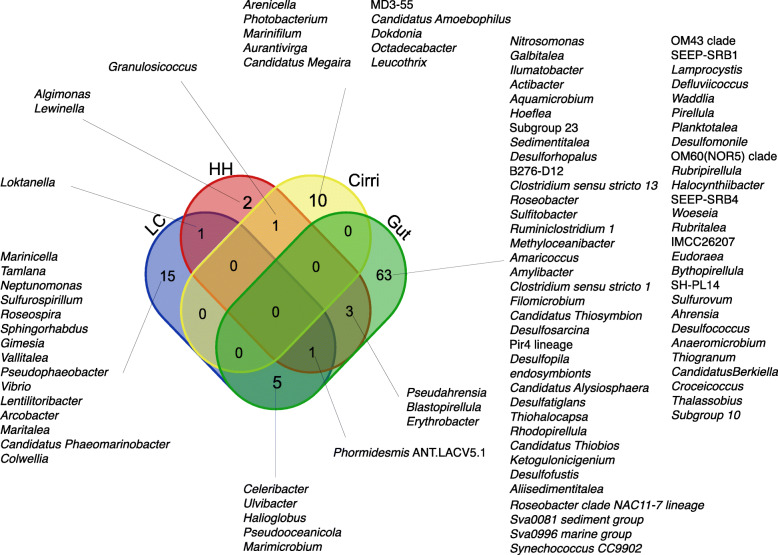
Distribution of genera from ASVs enriched in body tissue (gut and cirri), and microhabitat (LC and HH) based on species indicator analysis

Overall, microbiome diversity was higher in the gut, and body tissue explained a significant portion of variation in microbiome composition. The Shannon diversity index showed that the gut microbiome had higher microbial diversity than the cirri microbiome (t = − 4.3989, df = 19, *P <* .001) (Fig. 3a, Supplementary Table 5). We measured Bray-Curtis distance among samples and visualized using a PCoA plot (Fig. 3b). The dispersion test was not significant (F_19,1 =_ 2.0389, *P =* .18). This indicates that the groups’ dispersions were homogenous. PERMANOVA results showed microbiome composition between body tissue explained 16% of variation observed (F_19,1_ = 3.6495, *R*^*2*^ = 0.16, *P <* .001). We performed indicator species analysis to identify ASVs that were unique to each body part and driving the differences between cirri and gut microbiome. The indicator species analysis identified 207 ASVs that were characteristic to each body type (12 cirri; 195 gut) (Supplementary Figure 4 and Supplementary Table 6). These ASVs represented 83 genera (11 cirri; 72 gut) (Supplementary Figure 4). The indicator species analysis showed that taxa were non-overlapping between cirri and gut at the genus and ASV levels (Supplementary Figure 4). The top five genera with the highest mean abundance within the cirri microbiome were *Granulosicoccus*, *Leucothrix*, *Octadecabacter*, *Photobacterium*, and *Arenicella* (Supplementary Table 6). We found that of the ASVs that were gut specific, 64 were from the phylum Planctomycetes, and 98 were from Proteobacteria. The five gut specific ASVs with the highest mean abundance belonged to the genera *Halioglobusm*, *Pseudahrensia*, *Pir4 lineage*, *Sva0081 sediment group*, and *Sulfitobacter* (Fig. 4, Supplementary Table 6).

### Microbiome diversity and composition differences between microhabitats

Microhabitat explained a significant portion of variation within the cirri microbiome diversity (Shannon index) and composition (Bray-Curtis distance), but the gut microbiome did not follow this trend. Cirri microbiome diversity was higher in low and cold (LC) samples than high and hot (HH) samples (Shannon diversity index: t = 4.2301, df = 8, *P =* .003; Fig. 2a). Gut samples showed no significant differences in microbiome diversity between the LC and HH microhabitats using Shannon diversity index. With regard to composition, we found that differences in microhabitat (LC vs HH) explained a significant amount of variation in microbiome composition within the cirri (Bray-Curtis distance PERMANOVA: F_8,1_ = 2.4641, *R*^*2*^ = 0.23, *P =* .004, dispersion: F_8,1_ = 1.6892, *P =* .201), but not the gut (F_9,1_ = 1.234, *R*^*2*^ = 0.12, *P =* .093, dispersion F_9,1_ = 0.1394, *P =* .71). From the species indicator analysis, we found 33 ASVs that were microhabitat-specific based on species indicator analysis (Supplementary Figure 5, Supplementary Table 7). None of the ASVs associated with tidal height were shared between HH and LC microhabitats or with the cirri specific ASVs (Supplementary Table 6 and 7). *Granulosicoccus,* identified in the cirri, was specific to HH but not LC. Unlike the cirri, three microhabitats specific ASVs were shared between the gut, and HH and LC microhabitats. One ASV was shared between the gut and LC and two between gut and HH. When we looked at shared genera, we identified that microhabitat specific ASVs were associated with 28 genera (Fig. 4), two of which overlapped between LC and HH (*Loktenlla* and *Phormidesmis* ANT.LACV5.1). Nine genera that were specific to LC and HH were also specific to the gut (*Pseudahrensia*, *Blastopirellula*, *Erythrobacter*, *Phormidesmis* ANT.LACV5.1, *Celeribacter*, *Ulvibacter*, *Halioglobus*, *Pseudooceanicola*, and *Marimicrobium*).

### Predictive function of gut and cirri microbiome

**Fig. 5:**
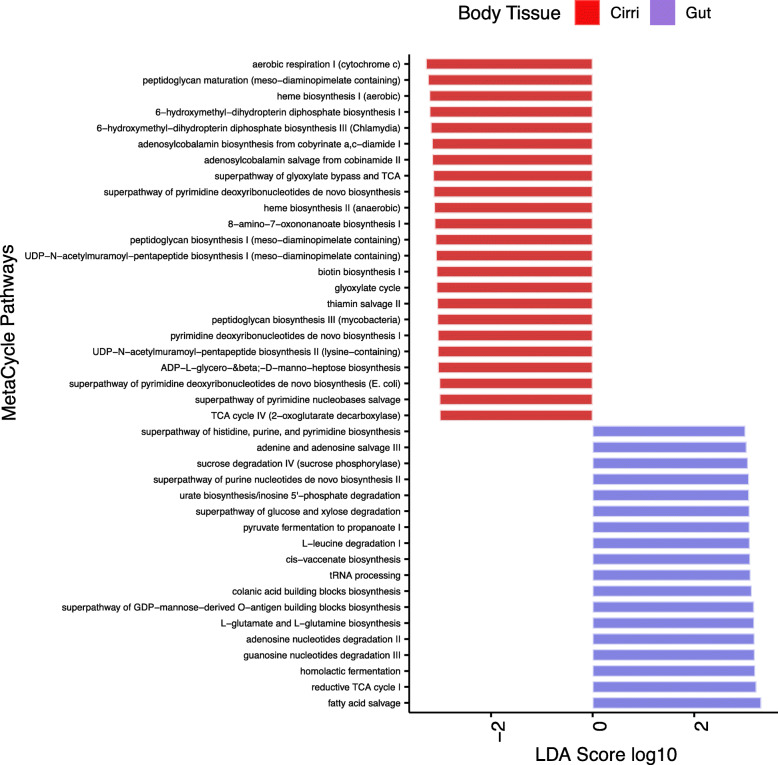
Predictive MetaCyc pathways based on ASVs enriched in cirri and gut based on indicator species analysis. The top distinct functions between body types determined by linear discriminant analysis (LDA) effect size (LEfSe) analysis

PICRUSt2 predicted 352 metabolic pathways from the 207 ASVs that were indicated to be specific to the gut or cirri (Supplementary Table 8). From LEfSe Analysis, 23 metabolic pathways were more abundant in the cirri, while 18 had higher abundance in the gut (Fig. 5, Supplementary Table 8). Both gut and cirri microbiomes were enriched with housekeeping pathways related to transcription and translation. The cirri microbiome was enriched with pathways associated with biotin synthesis, aerobic respiration, and thiamin salvage. The gut microbiome was enriched with nutrient associated metabolic pathways such as sucrose degradation, glucose and xylose degradation, and fatty acid salvage.

## Discussion

This study is the first to compare multiple tissue-specific microbiomes of the acorn barnacle *S. balanoides* and is a first attempt at understanding how the stresses experienced by barnacles inhabiting the extremes of the intertidal shape the microbiome. We sought to characterize the gut and cirri microbiomes of the barnacle *S. balanoides* and asked whether 1) the cirri, which interacts directly with the environment, had a distinct microbiome from the gut, and 2) whether location within the intertidal (LC vs. HH) influenced gut and cirri microbiome diversity and composition.

We found weak support for our first hypothesis that the microbiome of the gut would be similar to, or a subset of, the microbiome of the cirri. Our results show that the gut and cirri microbiomes shared similarities at the phylum level and shared 595 ASVs out of the unique 1477 ASVs observed (Fig. 2). However, the gut and cirri microbiomes were significantly distinct with regard to alpha diversity and composition measurements (Figs. 3 and 4), and body tissue explained 16% of the variation in microbiome composition. Moreover, the gut microbiome was not a subset of the cirri microbiome. Rather, the gut had more ASVs and taxa than the cirri (Figs. 2d and 3a). The compartmentalization of the cirri and gut microbiome observed within the barnacle is consistent with studies across several animals that show each body part has a distinct microbiome composition [35–37]. We surmise that the gut microbiome is distinct from, and more abundant and diverse than, the cirri’s simply due to the structural differences between the two body parts. The cirri are long filaments and do not offer microbes housing or protection against the elements (Fig. 1a and b). Moreover, the rapid movement of the cirri against water currents during feeding can result in the daily removal of microbes. In contrast to the filaments of the cirri, the gut of the barnacle is a single tube that is separated into fore, mid, and hind sections [15]. Guts of animals are often ideal habitats for microbes to inhabit and have consistently been shown to house a richer community when compared to other body parts [35, 38, 39]. Not only does the gut provide housing for microbes, it is also enriched with nutrients daily to feed those microbes [40]. Additionally, the barnacle gut is never directly exposed to the outside of the barnacle’s shell (Fig. 1b), and the only path of ridding the gut microbiome from the body is through excretion of feces [15]. The lack of environmental extirpation of the gut microbes may result in a consistent and stable microbiome when compared to the cirri.

We did not collect controls such as water samples from the water column or biofilm from the rock surface around the barnacle, so we could not compare and identify microbes that are host vs. environment associated within this system. To increase confidence that we characterized the barnacle microbiome, we compared similarities between the microbiome of the gut and cirri to that of the only other published *S. balanoides* study. The microbiome of *S. balanoides* in our study was similar in composition to Aldred and Nelson [4] at the phylum level and shared three of the top six taxa at the class level (Fig. 2a and b; Supplementary Table 4). Our analysis of Aldred and Nelson [4], showed that the top six phyla with the highest mean abundance were Proteobacteria (45%), Bacteroidetes (38%), Firmicutes (10%), Actinobacteria (2%), Bacteria unclassified (2%) and Planctomycetes (0.5%) (Supplementary Table 4). The top classes were Gammaprotebacteria (26%), Flavobacteria (25%), Alphaproteobacteria (11%), Sphingobacteria (8%), Clostridia (7%), and Bacteroidia (5%) (Supplementary Table 4). Notably, our dataset shows the same top phyla as that of Aldred and Nelson [4]. Despite differences at the class level, our datasets shared three of the top 6 classes (Alphaproteobacteria, Gammaproteobacteria, and Bacteroidia) (Supplementary Table 2, Supplementary Table 4). The shared taxa between *S. balanoides* from both studies offers confidence that we have accurately captured aspects of the microbiome. Furthermore, the shared taxa at the phylum level from distinct studies done on two different coasts of the North Atlantic Ocean (the United States and United Kingdom) suggests that microbiome taxa composition, at least at the phylum level, is to a certain degree species-specific.

*S. balanoides’* microbiome shares similar taxa at the phylum level with other crustaceans, but overall are more similar to other sessile invertebrates. The main commonality across crustaceans is that Proteobacteria is one of the predominant taxa. It is common for marine animals’ microbiomes to be inundated with Proteobacteria [39, 41, 42]. Proteobacteria is a ubiquitous clade that is found across several different marine environments [43]. Despite the similarity in Proteobacteria among crustaceans, their microbiomes differ compared to *S. balanoides*. The gut microbiome of lobsters and crabs have a high abundance of Tenericutes. In the Chinese mitten crab, *Eriocheir sinensis*, Tenericutes is the most abundant taxon in the gut microbiome [36, 44]. In shrimp, Firmicutes, Actinobacteria, and sometimes Fusobacteria were found to be among the top phyla in the intestinal microbiome [45–47]. Contrastingly, with regard to mean relative abundance, Actinobacteria was the sixth most abundant (4%); Tenericutes 0.1%, and Fusobacteria was almost non-existent (< 0%) in the gut microbiome of *S. balanoides* (Fig. 2a). Additionally, Planctomycetes (19% in gut) was among the top two phyla in the *S. balanoides* gut microbiome (Fig. 2a). However, Planctomycetes was not present amongst the top phyla of crab, lobster or shrimp.

The barnacle microbiome is similar to other sessile marine invertebrates. Dubé et al. [35] identified Proteobacteria, followed by the Bacteroidetes, Firmicutes, Planctomycetes, and many other unclassified bacteria as the top phyla among all tissues of the Black-lipped pearl oyster. A study of the gut microbiome of four freshwater mussels also identified similar top phyla, with Actinobacteria making up the fifth most abundant taxa [48]. Finally, Pierce and Ward [30] found that the top phyla of mussels and oysters consisted of Proteobacteria, Tenericutes, Verrucomicrobia, Bacteroidetes, Cyanobacteria, and Planctomycetes. If phylogeny played a more dominant role in shaping the *S. balanoides* microbiome, then there would be more similarities to other crustaceans than to sessile invertebrates. However, the similarity between *S. balanoides* and other sessile invertebrates suggests that the barnacle microbiome may be shaped primarily by life history, physiological, and ecological traits as opposed to phylogeny. Finer taxonomic resolution would likely alter the generality of these conclusions.

Bacteria that constitute the core microbiome are thought to be persistent symbionts and important to the host [41, 49, 50]. The gut microbiome had more core ASVs than the cirri. We identified nine ASVs that were present across 70% of cirri samples, and 71 ASVs across gut samples (Supplementary Figure 3). The higher number of core ASVs found within the gut suggests the gut may be able to form more stable and specific symbiotic associations. Only two core ASVs were shared between the gut and cirri. One was from the genus *Roseobacter*. Roseobacter is a widespread marine bacterial clade that displays a diversity of morphological and physiological features [43, 51]. The Roseobacter clade is often associated with coastal biofilms [51], and some of its members form a symbiotic relationship with several marine animals which has proven to be both helpful and harmful. In oysters, the Roseobacter clade is associated with juvenile oyster disease [52], while in scallop larvae, *Roseobacter* has shown to have probiotic effects such as protection against bacterial pathogens [53]. In addition to *Roseobacter*, *Granulosicoccus* is the most abundant genus in the cirri core microbiome. *Granulosicoccus* is mostly found associated with algae [54, 55]. This provides some evidence of consistent interaction of the cirri with the environment. Within the gut the two top genera were from *Aliiroseovarius* and *Desulforsarcina*. Species from *Aliiroseovarius* have been associated with increased thermal stress in corals and are generally found in seawater [56, 57]. Specifically, the species *Aliiroseovarius crassostreae* is associated with Roseovarius disease, which is responsible for the high mortality rate in juvenile eastern oysters [58]. *Desulforsarcina* are sulfate-reducing bacteria that are found in coastal marine waters [59]. The core microbiome found within the cirri and gut gives insight into the influence of the environment on the barnacle’s microbiome and the potential for diseases susceptibility. Further investigation into the putatively pathogenic *Roseobacter* and *Aliiroseovarius* during extreme temperatures is needed to deduce whether the genera are helpful, harmful or neutral to the barnacle.

Our second hypothesis that the cirri microbiome would be more labile than the gut between microhabitats was strongly supported. Cirri microbiome diversity and composition were significantly distinct between microhabitats (Fig. 3a and b). A comparison of the Shannon diversity index between cirri samples from LC vs. HH showed that the diversity of LC samples was higher (Fig. 3a). Additionally, microhabitat explained 23% of the observed variation in cirri microbiome composition. Contrastingly, the gut microbiome showed no significant differences in diversity or composition (Fig. 3a and b). The lack of microhabitat influence on the gut microbiome may be due to little or no environmental exposure. During feeding, the cirri are in direct contact with the environment, interacting with the pool of microbes within the water column (Fig. 1b). As such, we hypothesized and provided supporting evidence, that cirri microbiomes would vary as a function of the intertidal microhabitat. Notably, the results of other studies have shown that a host’s location within the intertidal influences microbiome diversity. In a study of the red algae, *Corallina officinalis*, Shannon diversity was higher in the upper shore when compared to mid and lower shore [32], whereas the sponge *Hymeniacidon heliophila* showed higher microbiome diversity in subtidal environments when compared to intertidal environments [31]. In our study, the cirri microbiome diversity was higher in LC than HH microhabitats (Fig. 3a). Since barnacles only extend their cirri when submerged underwater it is likely that the higher microbiome diversity of LC barnacles is due to more interaction with microbes within the water compared to their HH counterpart.

Taken together, our study and others [31, 32] suggest that a species’ microbiome responds in distinct ways to similar environmental variables, which will be an important consideration when implementing microbiome applications in conservation studies. These results hint at the intertidal environment having no influence on the gut microbiome. This finding motivates the hypothesis that, by not feeding and by keeping its shell tightly closed during high stress periods, the barnacle protects itself from the elements and also potentially is able to maintain a stable gut microbiome. Moreover, by only feeding during high tide the guts of barnacles in the upper and lower intertidal are likely exposed to similar microbial pools.

The ASVs that make up the barnacle gut and cirri microbiome are distinct from ASVs that are associated with a microhabitat. Results from our indicator species analysis identified the 207 (12 cirri; 195 gut) ASVs that were specific to gut or cirri, and only 3 of these overlapped with ASVs that were specific to a microhabitat (Supplementary Table 6). This small overlap indicates that most microbes that are associated with the gut and cirri are insensitive to microhabitat differences, providing evidence that there may be host selection of the cirri and gut microbiome. Despite evidence that there may be some host-selective effect, it is clear that biotic interactions in the environment play a strong role in shaping the cirri and gut microbiome of *S. balanoides*. The top ASVs specific to the gut (*Halioglobus*, *Pseudahrensia*, *Pir4 lineage*, *Sva0081 sediment group*, and *Sulfitobacter*) are commonly found in sediments and seawater [60–63]. Additionally, the two most abundant cirri specific ASVs *Granulosicoccus* and *Leucothrix* are mostly found associated with algae [54]. The presence of these environmentally associated taxa implies that the environment is playing an influential role in shaping the barnacle microbiome. Aldred and Nelson [4] showed that, when laboratory reared *S. balanoides* metamorphose from cyprid to juvenile in the lab, the dramatic shift of microbiome composition observed in the wild was diminished. This led the authors to the conclusion that environment played a key role in inoculating the microbiome of the barnacle. Habitat is a key factor in shaping the *S. balanoides* microbiome. Although we have provided evidence that our samples represent the barnacle microbiome, further investigations using host, sediment, and water controls are required to identify the extent to which hosts are filtering and maintaining a distinct microbiome from their environment.

The gut and cirri microbiome have distinct predictive metabolic pathways, and the metabolic pathways give some intuition about the role of the microbiome for suspension feeders within the intertidal. *S. balanoides* feeds on phytoplankton such as diatoms as well as harpacticoids, and detritus [15]. The gut microbiome consists of several nutrient extraction functions such as glucose and xylose degradation, sucrose degradation, and pyruvate fermentation suggesting that the gut microbiome has access to a rich carbon source (Fig. 5). These carbohydrate degradations and processing related functions are often observed in herbivores [64, 65]. The cirri microbiome is concentrated with functions associated with peptidoglycan biosynthesis, thiamin salvage and biotin biosynthesis (Fig. 5). Thiamine and biotin pathways are normally found in the healthy guts of animals [66, 67]. However, the source of these pathways in the cirri may be due to algal associated bacteria. Both biotin and thiamine are cofactors often associated with algal-associated bacteria and with algal blooms [68, 69]. The gut functions suggest that the microbiome may play a role in nutrition extraction within the barnacle. However, it is unclear if the functions observed are part of the gut microbiome or associated with transient bacteria that are passing through the gut. Thus, the extent to which these functions are useful to the host is unknown.

Although our study contributes knowledge of the microbiome of *S. balanoides*, it has several limitations associated with sample size, environmental controls, and the use of 16S rRNA gene to categorize function. The effect that we found between gut and cirri was highly significant, but future studies that focus on investigating the differences between the gut and cirri microbiome, should include a larger sample size and additional time points. We did not collect, nor did we perform sequencing on, adjacent sea water or sediment samples. However, we took precautions to properly store samples to decrease contamination and thoroughly rinsed samples prior to extraction to remove environmentally associated microbes. We also took the extra step to compare our results to another study of *S. balanoides* microbiome to identify coarse inaccuracies. Due to these limitations, we cannot conclude whether the microbiomes observed are specific sub-samples of the microbial diversity in the environment. Also, we cannot conclude if the microbiomes observed are resident or transient members of the microbiome community. However, we can confirm that the microbiome that we described consists of a bacterial community that is associated with multiple hosts, and there is consistency between gut and cirri samples across individuals collected at different locations.

## Conclusion

We described the gut and cirri microbiome of *S. balanoides* from microhabitats that experience upper (High Hot - HH) and lower limits (Low Cold - LC) microhabitats of the species temperature tolerance across two islands. We showed that the composition and abundance of the gut and cirri microbiome were distinct and responded uniquely to the environmental conditions of the microhabitats. Microbial diversity and composition of the cirri was influenced by microhabitat. The similarity between *S. balanoides* and other sessile invertebrates suggests that similarity in physiological, morphological, life-history traits, and abiotic and biotic factors strongly shape microbiome composition and diversity. Future research should focus on using more fine scale analyses of the microbiome, wider geographical ranges in conjunction with genetic resources, and information on the natural history of the system. Such studies will help elucidate the functions of the microbiome and the extent to which it can influence the fitness of the barnacle.

## Materials & methods

### Study site and ecological characteristics

We collected 12 *S. balanoides* individuals from two sites (*N* = 6 from each site) in the Damariscotta river in Maine, USA (43°53′47.1″N, 69°34′21.3″ W) in August 2015. The sites consist of two parallel islands, Farmer’s Island and Hodgson’s Island, which lie approximately 150 m apart on a region of high flow in the river (Fig. 1i). These two islands are of similar size and position in the river, both with a North-South orientation, and thus serve as replicates of each other. At each site, we collected barnacle individuals from the most thermally stressed and most thermally benign microhabitats in the intertidal. We sampled in sites previously characterized by Schmidt and Rand [23] and Nunez et al. [25]. Accordingly, the microhabitat in which barnacles experience the highest temperatures are those located on west-facing shores, lying near the upper edge of the tidal range. These sites have higher exposure to solar radiation during the day [23]. Consequently, they experience overall high levels of both mean thermal exposure (mean maximum T = 32.3 °C; Fig. 1e) and thermal variance (sd maximum T = 4.01 °C) (temperature data from [23]). Throughout this paper, the most stressful sites will be referred to as “HH” sites (for High and Hot). Conversely, the most benign microhabitats, those facing east and lying near the lower end of the intertidal, are referred to as “LC” (for Low and Cold). These low-stress habitats have the lowest levels of both mean thermal exposure (mean maximum T = 18.5 °C; Fig. 1e) and thermal variance (sd maximum T = 2.90 °C). On each island, our sampling was conducted in horizontal transects of ~ 20 m: LC sites were samples at vertical heights of ~ 0.10 m from sea level, HH sites were samples at vertical heights of ~ 1.8–2.0 m from sea level. In addition, we informed our collection based on tidal range information from the National Oceanic and Atmospheric Administration (NOAA) public tidal datasets [69]. We used data from the station 8416731 in Walpole, Damariscotta River, ME (Fig. 1d). For the month of August 2015, the mean low tide was 0.07 m, the lowest low tide was − 0.40 m and the highest low tide was 0.45 m (Fig. 1f). This signifies that, during the highest low tides, our LC populations were not exposed to air. Conversely, the mean high tide was 3.00 m, the lowest high tide was 2.50 m and the highest high tide was 3.64 m (Fig. 1f). As such, our HH sites were always exposed to air for at least 6 h every day. Overall, our experimental design includes samples from HH and LC microhabitats spanning the intertidal stress gradient on Farmer Island and Hodgson’s Island (Fig. 1i). Six individual barnacles were collected from each microhabitat (HH and LC) for a total of 12 gut and 12 cirri samples (Supplementary Table 1).

### Collection and storage

We collected barnacles that were exposed and not covered by algae. Additionally, we attempted to collect larger barnacles of the same size which had survived at least 1 year in the rocky shore and were reproductively mature. This was done to account for age-class discrepancies. We removed barnacles from rocks using flat head screwdrivers, and barnacles were transferred from rocks directly into sterile falcon tubes. To slow bacterial activity from time of collection to time of storage, barnacles were held in coolers on ice (< 10 °C) at the time of collection. We snap froze samples using liquid nitrogen within 3 h of collection, and samples were held at − 80 °C until extraction.

A total of 12 individuals were processed and subdivided to create 24 tissue samples (12 whole guts and 12 cirri) (Table 1). We dissected each of the 12 individuals to remove the cirri from the gut. Samples were processed one at a time. First, the barnacle’s prosoma was removed from the shell. We thoroughly rinsed the prosoma with sterile 1X PBS to remove loosely attached microbes. The rinsed individual was dissected in sterile Petri dishes. To avoid cross-contamination between gut and cirri, we first removed all cirri and placed them in a sterile Eppendorf tube containing 100 μl 1X PBS buffer. The gut was then carefully removed and placed in a separate sterile Eppendorf tube 100 μl 1XPBS buffer. Only completely intact guts were used for study. Samples were processed immediately after dissection. Each of our 24 samples, negative control (100 μl PBS buffer from extraction buffer stock), and a positive control containing pure culture *Escherichia coli* were homogenized with a hand pestle then vortexed for 10 mins in Vortex-Genie 2. We extracted microbial DNA from each sample using the Qiagen UltraClean® Microbial DNA Isolation kit following the manufacturer’s protocol using the alternative lysis step.

### 16S rRNA gene amplification and sequencing

We amplified the 16S rRNA gene region of the samples, negative control, and a positive control consisting of *Escherichia coli* DNA using the bacteria targeting v4v5 primers 518F CCAGCAGCYGCGGTAAN/926R CCGTCAATTCNTTTRAGT [70, 71] with and without sequencing facility adapter sequences. A two-step PCR protocol was carried out. The first step was performed using the following master mix recipe: 12.5 μl Phusion High Fidelity PCR Master Mix, 1.25 μl of each primer without adapters, 0.75 DMSO, and 1 μl template. DNA was amplified using the following thermocycler protocol: 1 min initial denaturation at 98 °C, 20 cycles of 10 s at 98 °C, 30 s at 57 °C, 30 s at 72 °C and final extension 5 min at 72 °C. An AMPure XP system PCR clean-up was carried out on PCR product. We performed a second PCR on the purified DNA using the following master mix protocol: 25 μl Phusion High Fidelity PCR Master Mix, 2.50 μl non- adaptor forward, 2.50 μl non-adaptor reverse primers, 2.50 DMSO and 1 ul purified DNA. Samples were amplified using the stated thermocycler protocol for ten cycles. PCR products were sent to the University of Rhode Island (URI) Sequencing Core for sequencing using the Illumina MiSeq 2 × 250 paired end reads (Illumina, Inc., San Diego, CA, United States). All raw sequences of 16S rRNA gene dataset obtained during this study were submitted to the Brown University digital repository (https://repository.library.brown.edu/studio/item/bdr:1093308/).

### Bioinformatics and statistical analyses

We quality checked and processed forward reads to create amplicon sequence variants (ASVs) using DADA2 [34]. Briefly, reads were truncated to 230 bp, and 17 bp primers were removed from the beginning of reads using the “trimLeft” function. We ran DADA2 with default settings for all other parameters. At this point, reads were observed as ASVs and all further analysis was done on ASVs. The taxonomic assignment of ASVs was done using DADA2 RDP naive Bayesian classifier and trained on the Silva version 132 training sets [72]. ASVs were assigned at the kingdom to genus level. Following the taxonomic assignment, we used a two-step method to identify and remove contaminants. First, Decontam was used to identify ASVs that were non-contaminants [73]. This was the manual’s suggested method for low-biomass samples for which contamination was a concern. ASVs that had a 0.5 probability threshold in the samples, and positive control when compared to the negative control, were defined as non-contaminants. After filtering contaminants, we removed ASVs taxonomically assigned to eukaryote, mitochondria, archaeal, chloroplast, and unidentified kingdom from the sequence table. Second, we checked to ensure our positive control only contained sequences taxonomically assigned to the genus *Escherichia*. After the contamination check, we removed positive and negative controls from the ASV table. To test if we captured the majority of the microbial community within samples, we created rarefaction curves and removed samples that did not plateau due to incomplete representation. We removed gut and cirri samples with less than 5000 ASVs, then rarified the sequence table to the minimum number of ASVs (10,372). At the end of the filtering process, we discarded three samples Cirri 12 HH, Gut 8 HH, and Cirri 10 HH, due to low ASV counts (Supplementary Table 1). After the removal of these samples, we had four cirri HH, six cirri LC, five gut HH, and six gut LC samples, totaling 21 samples (Table 1). All analysis was done using rarified count table. Due to the lack of environmental control, we compared the taxonomic abundance of our samples to only other *S. balanoides’* microbiome published study, Aldred and Nelson [4], to identify whether these two studies identified similar microbiome composition. Since the taxonomic composition of samples was not reported (it was not the goal of that paper), we calculated the percentage of phyla of *S. balanoides* from the biom table provided in the publication. This was done by reading the biom table into the *phyloseq* R package (version 1.30.0) in R and using the “taxa_summary” function to calculate composition [74].

### Microbiome diversity

To test if top phyla abundance were distinct between cirri and gut, we performed the Wilcoxon test from the *stat* package from R [75]. We investigated whether *S. balanoides* had a core microbiome by identifying the ASVs that were present in gut and cirri at 0.7 prevalence and 0% detection (ASV had to be present or absent) using the *microbiome* package in R [35, 76]. We used *phyloseq* to calculate the microbial diversity of each sample using the Shannon index [77]. To test whether microbial diversity between the gut and cirri microbiome was distinct, we performed a t-test from the *stats* package to evaluate significant differences in mean microbial diversity between body part. In order to identify if cirri microbial diversity was more responsive to microhabitat differences than the gut, we performed a t-test on the Shannon diversity of each body tissue between HH vs. LC.

### Microbiome composition

To test our hypothesis that the gut microbiome was a subset of the cirri microbiome, we used *phyloseq* to calculate Bray-Curtis distances on rarified sample counts [78]. We performed analysis on rarefied counts due to the sensitivity of the Bray-Curtis analysis to differences in library size. We created MDS PCoA plots to explore and visualize sample distances. We then performed permutational multivariate analysis of variance (PERMANOVA) using *adonis* function and analysis of multivariate homogeneity using the *betadisper* function from the *vegan* package in R, with body tissue as the grouping [79]. We calculated a *p*-value using a permutation test to evaluate whether there were differences in dispersion between groups. Both permutation tests were done with 999 permutations. Species indicator analysis was done using the “multipatt” function in the *indicspecies* R package, with body tissue as the grouping, to identify if any of the ASVs were characteristic of the gut or cirri [80]. ASVs with *P <* 0.05 were selected as indicator ASVs of each tissue type. To test our second hypothesis that cirri microbiome was more responsive than the gut microbiome to microhabitat differences, we performed PERMANOVA and multivariate homogeneity followed by a permutation test on each body tissue separately using microhabitat (LC vs. HH) as grouping. Finally, we wanted to identify the ASVs that were driving differences between the microbial communities found in different habitats. To identify driving differences between LC vs HH microhabitat, we performed another round of species indicator analysis on both cirri and gut using microhabitat as the grouping. Again, ASVs with *P <* 0.05 were selected as indicator ASVs for LC and HC.

### Predictive metabolic pathways

We predicted metabolic pathway abundances of ASVs associated with body part identified by the indicator species analysis using Phylogenetic Investigation of Communities by Reconstruction of Unobserved States (PICRUSt2) based on the MetaCyc metabolic pathway database [81, 82]. Briefly, we used the “picrust2_pipeline.py” function to run the full pipeline. This function performed sequence placement, hidden state prediction of genomes, metagenomic prediction, and pathway-level predictions. All ASVs within our dataset passed the nearest-sequenced taxon index (NSTI) cutoff (NSTI > 2). We then used the “add_descriptions.py” script to add the MetaCyc pathway data to predicted pathway abundances. We used Linear discriminant analysis Effect Size (LEfSe) to determine which MetaCyc pathways were more likely to explain differences between gut and cirri [83]. This was done by testing data for statistical significance, biological consistency, and effect size of the predicted pathways based on body type using LEfSe. An LDA threshold of 3.0 and significant cutoff < 0.01 were chosen as parameters for LEfSe.

## Supplementary Information

**Additional file 1:** **Supplementary Figure 1.** Rarefaction curves at 10,372 ASVs. Lines are labeled with sample names.

**Additional file 2:** **Supplementary Figure 2.** Boxplot showing mean relative abundance of the top six phyla. Wilcoxon rank sum test abundance showed that Actinobacteria (*P*=0.01) and Planctomycetes (*P*<0.001) are significantly higher in the gut when compared to the cirri.

**Additional file 3:** **Supplementary Figure 3.** Pie chart showing the mean relative abundance across sample type of ASVs found 70% prevalence. The innermost chart represents core ASVs found within cirri samples. The middle donut represents core ASVs found across gut samples. The outer donut represents core ASVs found across all individuals. Numbers are present for scale and represent axis for relative abundance.

**Additional file 4:** **Supplementary Figure 4.** Distribution of ASVs and associated genera enriched in body tissue from species-indicator analysis. A) Venn-Diagram showing ASVs enriched in each body tissue (gut vs. cirri). B) Venn-Diagram showing genera associated with ASVs enriched to each body tissue from A.

**Additional file 5:** **Supplementary Figure 5.** Distribution of ASVs and associated genera enriched in microhabitat from species-indicator analysis. A) Venn-Diagram showing ASVs enriched in each microhabitat (LC vs. HH). B) Venn-Diagram showing genera associated with ASVs enriched to each microhabitat from A.

**Additional file 6:** **Supplementary Table 1.** Mapping file and ASV counts.

**Additional file 7:** **Supplementary Table 2.** Taxa abundance at each level for gut and cirri samples.

**Additional file 8:** **Supplementary Table 3.** Taxa at 70% prevalence from core microbiome analysis.

**Additional file 9:** **Supplementary Table 4.** Abundance at phylum and class level from Aldred and Nelson [4].

**Additional file 10:** **Supplementary Table 5.** Alpha diversity metrics.

**Additional file 11:** **Supplementary Table 6.** Abundance of ASVs specific to gut and cirri based on species indicator analysis.

**Additional file 12:** **Supplementary Table 7.** Abundance of ASVs specific to LC and HH based on species indicator analysis.

**Additional file 13:** **Supplementary Table 8.** PICRUSt output.

## Data Availability

All raw sequences of 16S rRNA gene dataset obtained during this study were submitted to the Brown University Digital Repository Archive https://repository.library.brown.edu/studio/item/bdr:1093308/.
