# Supplementary figures and images for: Characterizing the cirri and gut microbiomes of the intertidal barnacle Semibalanus balanoides
Source: Anim Microbiome. 2020 Nov 13;2:41. doi: 10.1186/s42523-020-00058-0 (PMC7807441; doi:10.1186/s42523-020-00058-0)

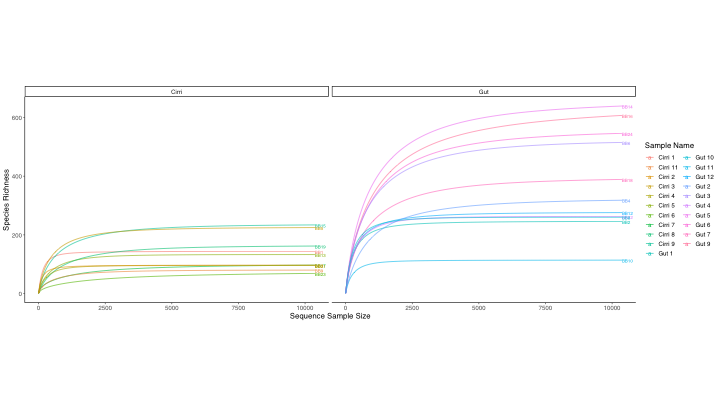

Supplement: Supplementary file 1 — Additional file 1: Supplementary Figure 1. Rarefaction curves at 10,372 ASVs. Lines are labeled with sample names. [file 42523_2020_58_MOESM1_ESM.tiff]

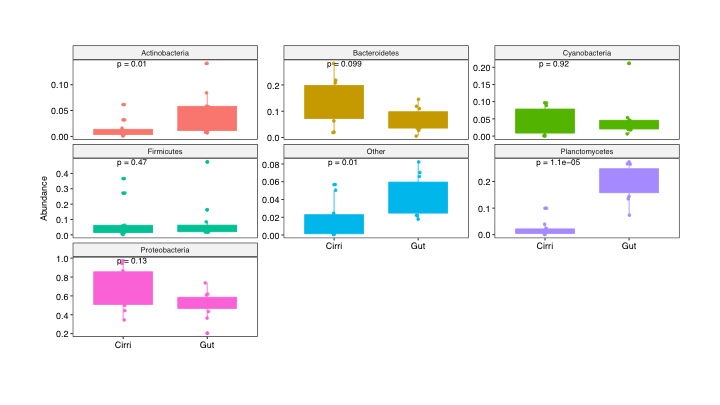

Supplement: Supplementary file 2 — Additional file 2: Supplementary Figure 2. Boxplot showing mean relative abundance of the top six phyla. Wilcoxon rank sum test abundance showed that Actinobacteria (P=0.01) and Planctomycetes (P<0.001) are significantly higher in the gut when compared to the cirri. [file 42523_2020_58_MOESM2_ESM.tiff]

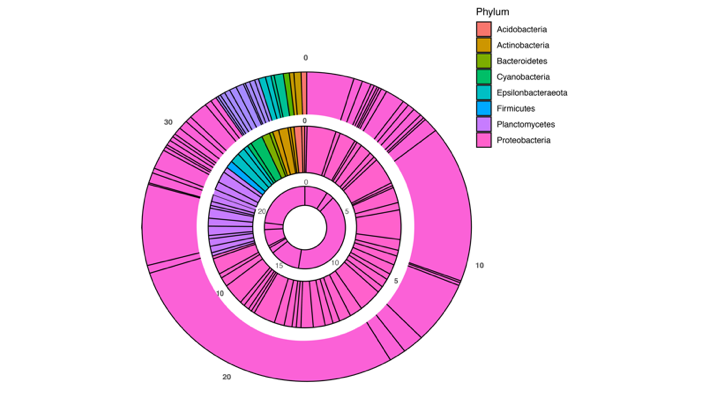

Supplement: Supplementary file 3 — Additional file 3: Supplementary Figure 3. Pie chart showing the mean relative abundance across sample type of ASVs found 70% prevalence. The innermost chart represents core ASVs found within cirri samples. The middle donut represents core ASVs found across gut samples. The outer donut represents core ASVs found across all individuals. Numbers are present for scale and represent axis for relative abundance. [file 42523_2020_58_MOESM3_ESM.tiff]

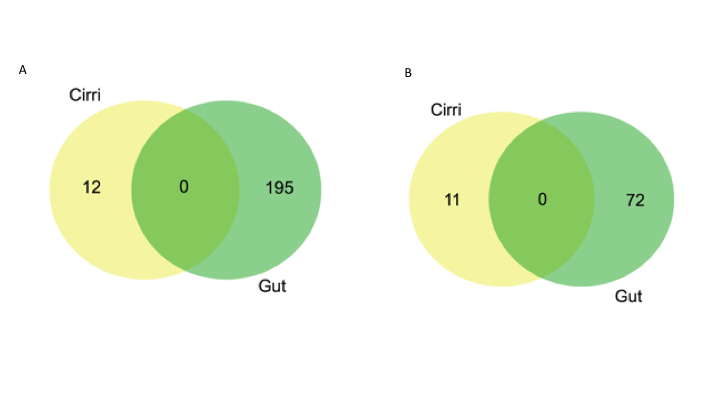

Supplement: Supplementary file 4 — Additional file 4: Supplementary Figure 4. Distribution of ASVs and associated genera enriched in body tissue from species-indicator analysis. A) Venn-Diagram showing ASVs enriched in each body tissue (gut vs. cirri). B) Venn-Diagram showing genera associated with ASVs enriched to each body tissue from A. [file 42523_2020_58_MOESM4_ESM.tiff]

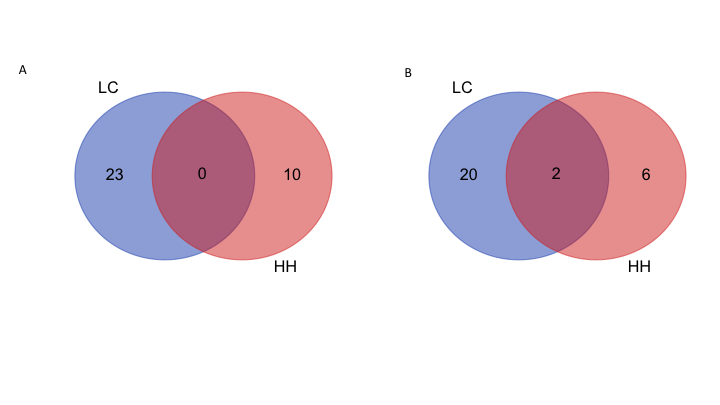

Supplement: Supplementary file 5 — Additional file 5: Supplementary Figure 5. Distribution of ASVs and associated genera enriched in microhabitat from species-indicator analysis. A) Venn-Diagram showing ASVs enriched in each microhabitat (LC vs. HH). B) Venn-Diagram showing genera associated with ASVs enriched to each microhabitat from A. [file 42523_2020_58_MOESM5_ESM.tiff]
